# Structural, antioxidant, and immunomodulatory activities of an acidic exopolysaccharide from *Lactiplantibacillus plantarum* DMDL 9010

**DOI:** 10.3389/fnut.2022.1073071

**Published:** 2022-12-08

**Authors:** Yan-yan Huang, Jia-min Wu, Wei-tong Wu, Jia-wei Lin, Yan-tong Liang, Zhen-zhen Hong, Xiang-ze Jia, Dong-mei Liu

**Affiliations:** ^1^College of Food Science and Engineering, Foshan University, Foshan, Guangdong, China; ^2^Guangdong Provincial Key Laboratory of Intelligent Food Manufacturing, Foshan University, Foshan, Guangdong, China; ^3^School of Food Science and Engineering, South China University of Technology, Guangzhou, Guangdong, China

**Keywords:** *Lactiplantibacillus plantarum* DMDL 9010, exopolysaccharide, structural characterization, antioxidant activity, structural features, immunomodulatory effect

## Abstract

This study investigated the structural, antioxidant, and immunomodulatory activities of acidic exopolysaccharide (EPS-LP2) isolated from *Lactiplantibacillus plantarum* DMDL 9010. EPS-LP2 is composed of fucose (Fuc), arabinose (Ara), galactose (Gal), glucose (Glc), mannose (Man), and D-fructose (Fru) with a molar ratio of 0.13: 0.69: 8.32: 27.57: 62.07: 0.58: 0.46, respectively. Structural analysis of EPS-LP2 exhibited a smooth irregular lamellar surface, rod-like structure with swollen ends and slippery surfaces, and good thermal stability. Based on the methylation and NMR analysis, sugar residues including t-Man*p*, t-Glc*p*, 2-Man*p*, 6-Gal*p*, 6-Glc*p*, and 4-Glc*p* were found to exist in EPS-LP2. In the 50∼400 μg/ml range, EPS-LP2 showed negligible neurotoxicity to RAW264.7 cells. Moreover, EPS-LP2 could protect RAW264.7 cells from oxidative injury by lowering the generation of reactive oxygen species (ROS), malondialdehyde (MDA), and the secretion of lactate dehydrogenase (LDH). In contrast, an increase in superoxide dismutase (SOD), catalase (CAT), glutathione peroxidase (GSH-Px), and the concentrations of glutathione (GSH) were observed. Immunoreactivity assays showed that EPS-LP2 could suppress the expression of NO, tumor necrosis factor-α (TNF-α), and interleukin 6 (IL-6) and inhibit the activation of the mitogen-activated protein kinase (MAPK)/nuclear factor-κB-gene binding (NF-κB) cell pathway. Conclusively, EPS-LP2 could be a potential natural antioxidant and immunomodulatory agent in functional foods and medicines.

## Introduction

Probiotics are beneficial microorganisms for humans or animals, which are widely used in developing healthcare products and clinical medicine. They could avoid or reduce diseases such as diarrhea and colitis by forming an intestinal barrier (1–3). The more common ones include *Bifidobacterium*, *Lactobacillus*, and *facultative anaerobe*. Exopolysaccharides (EPS) are mainly produced by plants, algae, fungi, and bacteria [Especially lactic acid bacteria (LAB)]. For LAB, the outer layer of EPS protects from desiccation and phage attacks, potentially valuable for forming biofilms. EPS-derived LAB has multiple applications in the food industry, usually used as thickeners, gelling agents, and emulsifiers to improve the shelf life and quality of packaged food materials. EPS produced by food-grade LAB is generally acknowledged as safe and can be used as a living alternative to EPS from plant and animal sources. Nowadays, more attention has been paid to the therapeutic applications of EPS from LAB (4–6). These studies mainly focused on yogurt starters with a technical interest, such as *Lactobacillus delbrueckii, Lactococcus lactis*, and *Lacticaseibacillus casei* (7).

*Lactiplantibacillus plantarum* is usually isolated from food and considered a probiotic due to some specific characteristics. Free radicals and reactive oxygen species (ROS) play a significant role in cancer, liver cirrhosis, fatty liver, and other diseases. However, ROS’s excessive or insufficient free radical scavenging capacity *in vivo* will lead to cell membrane damage and promote cell death (8). Intake of antioxidants benefits human health because they impede the level of ROS and free radicals (9). The immunoregulatory activity of EPS in LAB is mainly due to the production of interleukin 6 (IL-6), interleukin 1β (IL-1β), tumor necrosis factor-α (TNF-α), and phagocytosis of macrophages (10). EPS-K4 from *Bacillus amyloliquefaciens* DMBA-K4 produce TNF-α and IL-10 and decrease the levels of virulent substances such as endotoxin, diamine oxidase, and D-lactic acid in serum to stimulate mucin secretion to maintain the integrity of the gut barrier (1). EPS produced by LAB has been widely used to improve yogurt’s rheology, texture, and taste (11). Previous studies have found that EPS extracted from fungi have similar effects (12). Therefore, EPS produced by LAB can potentially be used in food additives or functional food ingredients with health and economic benefits.

The study aimed to isolate, the EPS from *L. plantarum* DMDL 9010. After that, the EPS was purified, identified, and analyzed to explore its antioxidant and immunomodulatory activities. These outcomes can help us to identify the potential applications of EPS in dairy and other food industries. Also, provide us with some basis for linking the inherent attributes of EPS with its assumed future health benefits.

## Materials and methods

### Strain and exopolysaccharides production

LP9010 (CGMCC No. 5172), with the genome sequence accession numbers CP063986–CP063988, was separated from traditionally fermented pickles (13). LP9010 was cultivated to produce EPS in MRS broth (Guangdong Huankai Co., Ltd., Guangdong, China) at 37°C for 18 h. The collected fermentation supernatant was concentrated by a rotary evaporator (RE-2000A, Shanghai Ya Rong, Shanghai, China).

### Isolation, purification, and molecular weight determination of exopolysaccharides

**FIGURE 1:**
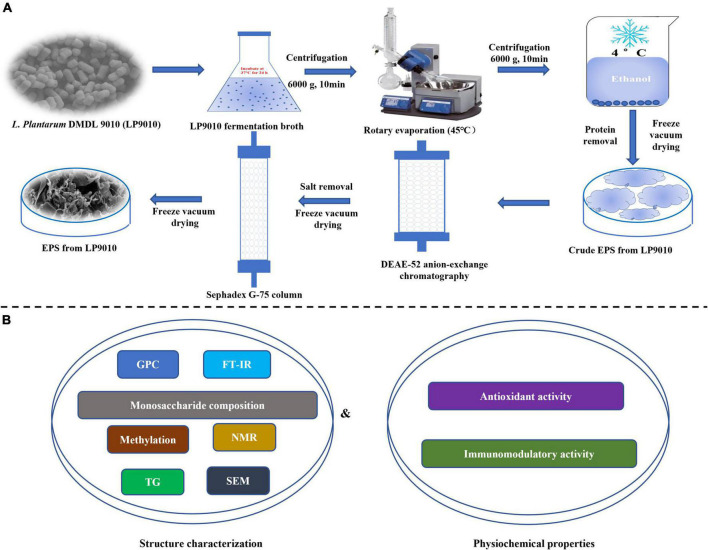
The procedure of the whole experiment. **(A)** The investigations of isolation, purification, and molecular weight determination of exopolysaccharides (EPS). **(B)** The experiments of structure characterization and biological.

The isolation and purification procedures were performed according to the method of Kuang et al. (1), with minor changes (Figure 1A). Sevag reagents precipitated ethanol (4°C, 24 h) and deproteinized to acquire crude EPS. Then, the crude lyophilized EPS was dissolved in pure water at 20 mg/ml concentration and filtered by a 0.22 μm filter. The EPS solution was eluted with gradient concentrations of sodium chloride solutions (0, 0.1, 0.3, and 0.5 M) at a flow rate of 1.5 ml/min by dicthylaminoethyl (DEAE)-Cellulose-52 anion exchange column (2.6 cm × 40 cm, GE Healthcare, Beijing, China). The eluate (10 ml/tube) was gathered and analyzed by the phenol-sulfate method. The crude EPS-LP2 was purified by Sephadex G-75 column (1.6 cm × 50 cm, GE Healthcare, Beijing, China) as described by Huang et al. (12) and eluted with pure water (0.5 ml/min). The eluent from each tube was gathered and analyzed. The purified EPS-LP2 with the highest absorption peak was collected and freeze-dried for further analysis.

The molecular weight of the purified EPS-LP2 was determined by adopted the method of Huang et al. (12) by high-performance gel permeation chromatography (HPGPC) with a TSK-gel-G3000*_*PWXL*_* column (0.78 cm × 30 cm, Tokyo, Japan). The diverse dextran standards from 5.0 to 670 kDa were used to calculate the molecular weight.

### General characteristics of the exopolysaccharides-LP2 from LP9010

To evaluate the general properties of the EPS-LP2 ultraviolet (UV) spectrum, Fourier-transform infrared spectroscopy (FT-IR), scanning electron microscopy (SEM), and thermogravimetric (TG) analyzer were used. For the UV absorption spectrum of EPS-LP2, speedily scanned the UV spectrum with a wavelength ranging from 200 to 400 nm. For FT-IR analysis, the EPS-LP2 was mixed with KBr powder, then pressed into a 1 mm pellet, and analyzed on a FT-IR spectrometer (Nicolet IS50, Thermo Fisher Scientific, MA, USA). The IR spectrum ranged from 500 to 4000 cm^–1^ with 4 cm^–1^ resolution. For microcosmic morphology, EPS-LP2 (5 mg) was plated with a layer of approximately 10 nm-thick gold and observed by SEM (EM-30 Plus, COXEM, Silicon Valley, South Korea) at 20 kV. The EPS-LP2 (25 mg) was measured by the TG Analyzer (TG209F1, NETZSCH GmbH, Selb, Germany) in the nitrogen atmosphere with a heating rate of 10°C/min (25–800°C) at a flow rate of 10 ml/min.

### Monosaccharide composition, methylation, and nuclear magnetic resonance analysis of exopolysaccharides-LP2

The structure of EPS-LP2 was analyzed for monosaccharide composition, methylation and nuclear magnetic resonance (NMR) analysis, as shown in Figure 1B. For monosaccharide composition, 5 mg of EPS-LP2 was hydrolyzed (121°C for 2 h) with 4 ml of trifluoroacetic acid (TFA) (2 mol/L). TFA was dried with a blow of nitrogen, cleaned with methanol, and dried the methanol with a blow 2–3 times repeatedly. The sample was dissolved in sterile water and filtered through a 0.22 μm filter for high performance liquid chromatography (HPLC) (ICS5000+, Thermo Fisher Scientific, MA, USA) and Dionex™ CarboPac™ PA10 column (250 × 4.0 mm, 10 μm). The mobile phase comprised solution A (distilled water) and solution B (100 mM NaOH). The process of gradient elution was as follows: 0→30.0→30.1→45→45.1→60 min, and correspondent solvent B: 2.5→20→40→40→2.5→2.5% (flow speed = 0.5 ml/min).

According to the previous study (12, 14), the methylated EPS-LP2 continued to be acetylated following acid hydrolysis. Then, the methylation derivative was measured by GC-MS (7890A-5977B, Agilent Technologies, CA, USA) equipped with a quadrupole mass spectrometry system with a DB-5 ms fused silica capillary column (0.25 mm × 0.25 μm × 30 m). The carrier gas was high-purity helium with a 10:1 split ratio. The initial temperature of the column incubator was 140°C for 2 min, and the temperature program was 230°C at 3°C/min for 3 min.

For NMR analysis, 60 mg dried EPS-LP2 was dissolved in 0.6 ml D_2_O. After that, it was repeatedly lyophilized and dissolved three times for further measurement by an NMR spectrometer (AVANCE III HD 600, Bruker, Karlsruhe, Germany) of the ^1^H and ^13^C spectra.

### Cellular antioxidant activity

#### Cell culture and cell viability test

Following Zhou’s approach (15), RAW264.7 cells were cultivated in Dulbecco’s modified eagle medium (DMEM, including 10% fetal bovine serum and 1% streptomycin-penicillin) and incubated at 37°C in an incubator (MCO-18 AC, Panasonic, Osaka, Japan) with 5% CO_2_. MTT assay was applied to identify the cytotoxic effect of EPS-LP2 on macrophages (16). Cells were firstly treated with EPS-LP2 solution at different concentrations (0, 50, 100, 200, and 400 μg/ml, 100 μL) for 2 h. Cells were then stimulated with DMEM containing 1 μmol/L H_2_O_2_ as a blank and treated with vitamin C (Vit C) (100 μg/ml) as a positive control. MTT (10 μL) was included in each cell and incubated for 2 h at 37°C in an incubator with 5% CO_2_ incubator. The sample absorbance values were measured at 570 nm with a microplate reader (SMR60047, USCNK).

#### Intracellular reactive oxygen species

The DCFH-DA method was used to detect the standard of ROS in RAW264.7 cells (17). The cells were first treated with different concentrations (0, 50, 100, 200, and 400 μg/ml, 100 μl) of EPS-LP2 solution for 2 h, then stimulated with 1 μmol/L H_2_O_2_ for 24 h. Following this, at room temperature, the cells were cleaned with trypsin (0.25%) until the cells were circular, and then the digestion was stopped by adding DMEM complete medium. Cells were collected in a cell tube and then DCFH-DA (10 μmol/L) was added. In the following, the samples were incubated for 20 min at 37°C, centrifuged, and cells were cleaned with DMEM complete medium to eliminate the residual DCFH-DA. Cells were resuspended by adding PBS and then analyzed within 1 h using a FACSCalibur flow cytometer (FACSCalibur, NJ, USA) to determine the ROS levels. DMEM was conducted as a blank control and Vit C (100 μg/ml) as a positive control.

#### Determination of the cell antioxidant status

The cells were first incubated with different concentrations of EPS-LP2 solution (0, 50, 100, 200, and 400 μg/ml, 100 μl) for 2 h. Then cells were incubated with 1 μmol/L of H_2_O_2_ for 24 h. After that, collected and centrifuged the cell cultures. The upper clear layer was assayed for the effect of polysaccharide solution on the cellular secretion of malondialdehyde (MDA), superoxide dismutase (SOD), glutathione (GSH), glutathione peroxidase (GSH-Px), catalase (CAT), and lactate dehydrogenase (LDH). DMEM was conducted as the blank control and Vit C (100 μg/ml) as a positive control.

### Immunomodulatory activity

#### Cell survival test

The cell viability of EPS-LP2 against RAW264.7 cells was measured by the CCK-8 method. RAW264.7 cells at the logarithmic growth stage were inoculated at 1 × 10^6^ cells/ml in a 96-well plate at 100 μL per well. After incubation with fresh DMEM medium for 24 h at 37°C in a 5% CO_2_ incubator, the upper supernatant was discarded. Then DMEM culture medium with different concentrations (0, 50, 100, 200, and 400 μg/ml, 100 μL) of polysaccharides was added to each well, and incubation was continued for 24 h (normal culture medium was used as blank control). After discarding the drug-containing medium, the cells were incubated with 10% CCK-8 solution for 2 h. Cell viability was determined by measuring the absorbance at 450 nm. Cell viability is calculated by the formula;


(1) $$\mathrm{Cell}\mathrm{viability}\left(\%\right)=\frac{A\,2}{A\,1}\times 100$$

A1 is the absorbance value of the blank sample and A2 is the absorbance value of the test sample.

#### Measurement of nitric oxide and cytokines

The cells were first treated with different concentrations of EPS-LP2 (0, 50, 100, 200, and 400 μg/ml, 100 μl) solution for 2 h, and then incubated by stimulating with lipopolysaccharid (LPS) (1 μg/ml) for 24 h. After that, centrifugation was done at 10,000 *g*, 25°C, for 10 min, and the nitric oxide (NO) assay kit determined the NO content. The ELISA kit determined IL-6 and TNF-α concentrations in the supernatants (Nanjing, China). DMEM was used as a blank, and DXMS (100 μg/ml) was used as a positive control.

#### Analysis of western blotting

The expression levels of phosphorylated protein-38 (p-p38), phosphorylated c-jun N-terminal kinase (p-JNK), phosphorylated extracellular regulated protein kinases (p-ERK), phosphorylated protein-65 (p-p65), and phospho-inhibitory subunit of NF-κBα (p-IκB-α) were measured by Western blot in RAW264.7 cells (18). β-actin expression levels were used as a standard internal control.

### Statistical analysis

All data were analyzed and drawn *via* GraphPad Prism (version 8.0.1). The results are represented as mean ± standard deviation, and analysis of variance was carried out by Tukey’s test (*p* < 0.05). Significant differences among groups were evaluated by one-way analysis of variance (ANOVA) with a level of significance of *p* < 0.05 compared with the control group, and *p* < 0.05 compared with the LPS-induced group.

## Results

### Production, separation, and purification of exopolysaccharides from LP9010

**FIGURE 2:**
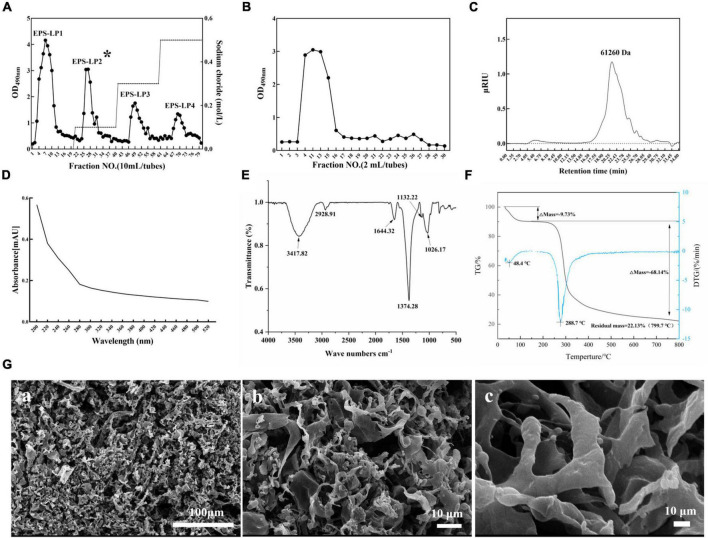
Production, separation, purification, and general properties analysis of exopolysaccharides (EPS)-LP2 from LP9010. **(A)** Chromatograms of EPS purified by DEAE cellulose-52 anion exchange column and **(B)** Sephadex G-75 column, **(C)** high-performance gel permeation chromatography (HPGPC) spectrum of EPS-LP2, **(D)** ultraviolet (UV), **(E)** fourier-transform infrared spectroscopy (FT-IR) spectra, **(F)** thermogravimetric (TG) analysis curve of EPS-LP2, and **(G)** scanning electron microscopy (SEM). (a) 100 μm, (b) 10 μm, and (c) 10 μm of extrapolysaccharide (EPS)-LP2.

The 0.5641 g crude EPS obtained from LP9010 was fractionated on a DEAE cellulose-52 anion exchange column. Figure 2A identified four peaks labeled EPS-LP1 to EPS-LP4 from left to right, and among these, EPS-LP1 had a higher absorption peak. Research results have shown that EPS-LP1 also exhibits promising antioxidant and immunological effects (data not published). Large amounts of crude EPS-LP2 were then collected and purified using a Sephadex G-75 column (Figure 2B). The purified EPS-LP2 (0.2392 g) yielded 42.40% *via* dialysis and freeze-drying. Earlier, Lian et al. (19) demonstrated that the molecular weight of EPS could affect its biological activity; high molecular weight appeared more efficient than low molecular weight. Furthermore, HPGPC was performed to determine the molecular weight of EPS-LP2. The singlet of EPS-LP2 was observed (Figure 2C), indicating a highly homogeneous structure for EPS-LP2.

### General properties analysis of exopolysaccharides-LP2

The purity of EPS-LP2 was examined using a UV-vis spectrometer. According to a previous study (20), no absorption peak at 260 or 280 nm (Figure 2D) was found in the spectra of purified EPS-LP2, indicating the absence of nucleotide or protein contamination. Fourier transforms infrared (FTIR) experiment was conducted to investigate the main functional groups of EPS-LP2. As shown in Figure 2E, the broad stretching peak at 3417.82 cm^–1^ belongs to the hydroxyl group (12). The peak at 2928.91 cm^–1^ is due to the asymmetric C and H stretching vibrations of the aliphatic CH_2_ group, indicating organic matter such as sugars (21). The peak structural characterization and biological activity of the extracellular polysaccharide of EPS-LP2 at 1644.32 cm^–1^ was similar to that of mannose or galactose (21). The vibration at 1374.28 cm^–1^ may be attributed to the symmetric stretching of the carboxyl group (1). The polysaccharides are present because of a strong absorption peak at 1026.37 cm^–1^ (22). The absorption peak at 1026.37–1132.22 cm^–1^ indicates the possible presence of a furan ring in the structure of EPS-LP2 (12).

Scanning electron microscopy performed the morphological studies, and the surface of EPS-LP2 showed diverse structures (Figure 2G). With increasing magnification, some of them could be observed as irregular lamellar structures with smooth surfaces and some tissues adhering to each other, while some parts showed rod-like designs with swollen ends and smooth surfaces [Figure 2G (a)]. The highly branched network of EPS- LP2 may be favorable for its application in food to improve its water-holding capacity and viscosity (23). Thermal stability is significant to commercializing polysaccharides. The thermal stability was determined by TG analysis. The results showed that EPS-LP2 was degraded in two consecutive weight-loss phases (Figure 2F). The initial 9.73% weight loss at 188.8°C was mainly due to water evaporation. In contrast, 68.14% decomposition was observed at 188.8∼ 800°C, indicating that EPS-LP2 exhibits layered thermal stability, which implies potential practical applicability in the dairy industry.

### Monosaccharide composition, methylation, and nuclear magnetic resonance analysis of exopolysaccharides-LP2

**FIGURE 3:**
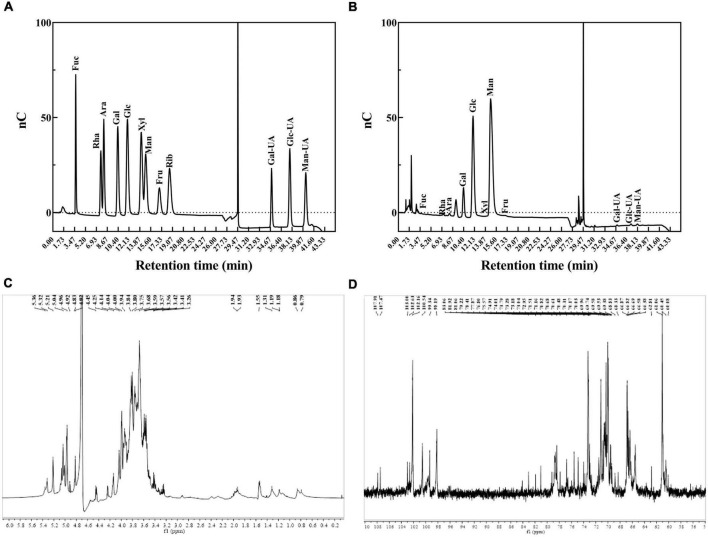
Monosaccharide composition analysis and nuclear magnetic resonance (NMR) spectra of exopolysaccharides (EPS)-LP2. **(A)** HPLC analysis of monosaccharide composition of monosaccharide standard product (Fuc, fucose; Rha, rhamnose; Ara, arabinose; Gal, galactose; Glc, glucose; Xyl, xylose; Man, mannose; Fru, fructose; Rib, ribose); **(B)** HPLC analysis of monosaccharide composition of EPS-LP2; **(C)** ^1^H nuclear magnetic resonance (NMR) spectrum; **(D)** ^13^C NMR spectrum.

The monosaccharide composition analysis showed that EPS-LP2 was mainly composed of mannose. The molar ratio of each monosaccharide was fucose (Fuc): arabinose (Ara): galactose (Gal): glucose (Glc): mannose (Man): D-fructose (Fru): and galacturonic acid (Gal A) = 0.13: 0.69: 8.32: 27.57: 62.07: 0.58: 0.46, indicating that EPS-LP2 was a heteropolysaccharide (Figures 3A,B). The glycosyl residues’ linkage was determined by methylation analysis (12). However, due to the presence of many miscellaneous signals in the GC-MS data, it was difficult to complete the quantitative analysis of the glycosidic bond, so the methylation result was not provided here. While the glycosidic linkages of EPS-LP2 including t-Man*p*, t-Glc*p*, 2-Man*p*, 6-Gal*p*, 6-Glc*p*, 4-Glc*p* were detected. The ^1^H NMR spectra were utilized to investigate the composition of glycosidic bonds within EPS-LP2 (Figure 3C). No resonances were observed between δ 6 and 8 ppm, revealing that EPS-LP2 is highly pure and free of phenolic or ferulic impurities. The anomeric hydrogen distribution ranged between 4.8 and 5.4 ppm, indicating that EPS-LP2 contained α- and β- glycosidic bonds. ^13^C NMR spectra could reflect the residual number of polysaccharides in the sample. In addition, the number of polysaccharide residues and their associated configurations could be analyzed and determined by the peak number of anomeric carbons with chemical shifts between 95 and 110 ppm. The ^13^C NMR spectra of EPS-LP2 are displayed in Figure 3D. The anomeric carbons were found at δ 103.00, 102.63, 102.16, 100.54, 99.34, and 98.19 ppm. The strongest peak was detected at δ 102.16 ppm, indicating that EPS-LP2 contained six kinds of glycosidic bonds, consistent with the methylation results. More detailed information about the locations and sequences of the six glycosidic linkages will be clarified in the future.

### Effect of exopolysaccharides-LP2 on cellular enzymatic antioxidant system

**FIGURE 4:**
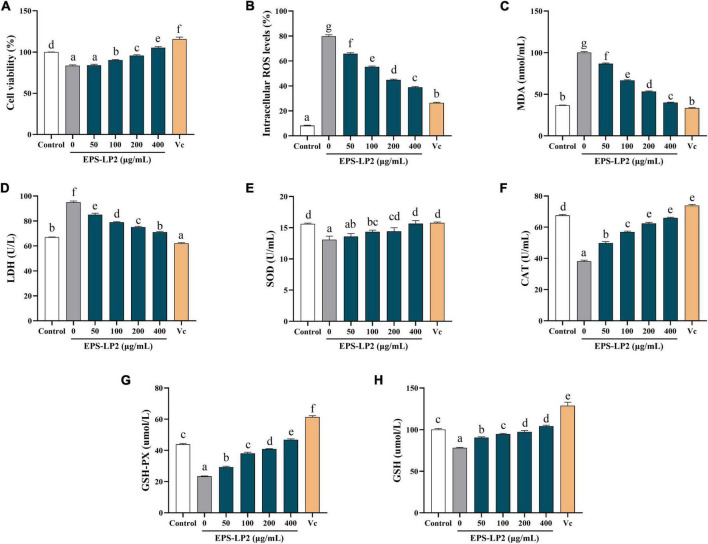
Effects of exopolysaccharides (EPS)-LP2 on **(A)** cell viability, the levels of **(B)** reactive oxygen species (ROS), **(C)** malondialdehyde (MDA), **(D)** lactate dehydrogenase (LDH), **(E)** superoxide dismutase (SOD), **(F)** catalase (CAT), **(G)** glutathione peroxidase (GSH-Px), and **(H)** GSH in RAW264.7 cells induced by H2O2. Different letters represent different significance from the control group (*p* < 0.05).

As illustrated in Figure 4A, EPS-LP2 strengthened the viability of H_2_O_2_-induced RAW264.7 cells compared to the blank and negative controls, indicating that EPS-LP2 was no toxicity to RAW264.7 cells from 50 to 400 μg/ml concentration.

#### Effect of exopolysaccharides-LP2 on reactive oxygen species, malondialdehyde, and lactate dehydrogenase production

The changes in ROS, MDA, and LDH contents could reflect the level of cell damage. As presented in Figure 4, levels of intracellular ROS (Figure 4B), MDA (Figure 4C), and LDH contents (Figure 4D) were significantly higher in RAW 264.7 cells than in non-hydrogen peroxide-treated cells. Treatment with H_2_O_2_ (110.87 ± 4.52 nmol/ml), indicated that the cells were in an oxidative stress state. In the existence of EPS-LP2, ROS, MDA, and LDH contents decreased in a dose-dependent manner. Moreover, the contents of ROS, MDA, and LDH were relatively equivalent to the Vit C positive control at EPS-LP2 concentration of 400 μg/ml. The present results demonstrated that EPS-LP2 could be protected against oxidative damage.

#### Effect of exopolysaccharides-LP2 on cellular enzymatic antioxidant system

SOD, CAT, and GSH-Px are all extremely important antioxidant enzymes that could perform vital functions in oxidative and antioxidant homeostasis in the human body. As shown in Figures 4E–G, the activities of all three antioxidant enzymes were remarkably decreased by treatment with hydrogen peroxide. After being treated with EPS-LP2 of different concentrations, the activities of intracellular antioxidant enzymes significantly improved (*p* < 0.05). At EPS-LP2 concentration of 400 μg/ml, the activities of intracellular SOD, GSH-PX, and CAT were 15.648 ± 0.49 U/ml, 46.829 ± 0.56 μmol/l, 65.982 ± 0.45 U/ml, respectively. The results indicated that EPS-LP2 could protect RAW264.7 cells from H_2_O_2_-induced oxidative stress by enhancing the SOD (Figure 4E), CAT (Figure 4F), and GSH-Px (Figure 4G) activities.

#### Effect of exopolysaccharides-LP2 on the cellular non-enzymatic antioxidant system

Other essential antioxidant substances (e.g., GSH) also perform a critical function in the body’s antioxidant balance. Thus, this experiment was performed to measure the changes in intracellular GSH values to determine the extent of cell damage. As shown in Figure 4H, the presence of EPS-LP2 decelerates the degree of reduction of antioxidant substances, and the intracellular GSH content reached 104.110 ± 1.24 μmol/l at an EPS-LP2 concentration of 400 μg/ml, indicating that EPS-LP2 also enhanced the non-enzymatic antioxidant systems of the cells.

### Effect of exopolysaccharides-LP2 on cell vitalization

**FIGURE 5:**
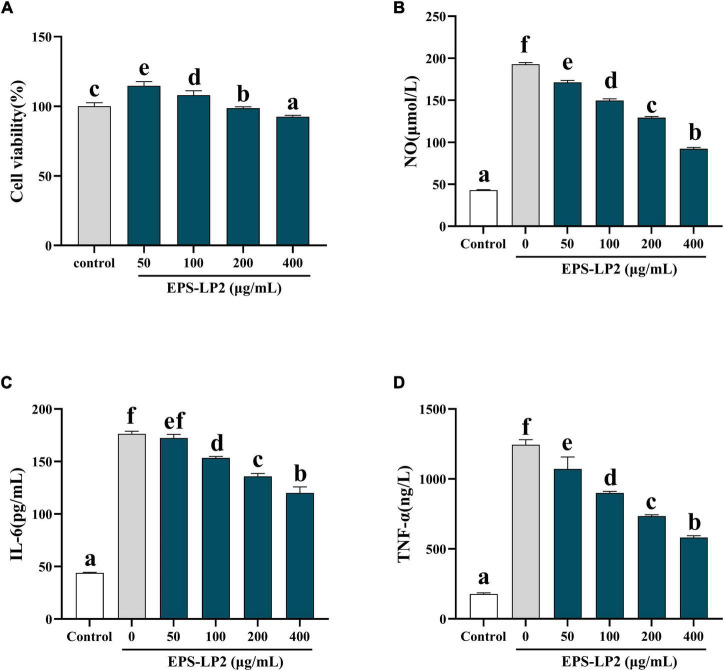
Effects of exopolysaccharides (EPS)-LP2 on **(A)** cell viability, **(B)** nitric oxide (NO) production, expression of **(C)** interleukin 6 (IL-6), **(D)** tumor necrosis factor-α (TNF-α) in RAW264.7 cells induced by lipopolysaccharid (LPS). Different letters represent different significance from the control group (*p* < 0.05).

Figure 5A indicates the effect of EPS-LP2 on LPS-induced cell survival rate. The results showed that EPS-LP2 did not influence the viability of the cells in the range of 50–400 μg/ml compared to the control and negative samples. Five concentrations of 0, 50, 100, 200, and 400 μg/ml of EPS-LP2 were selected for cell immunization experiments, and a concentration of 200 μg/ml was chosen for immunoblot analysis.

#### Effect of exopolysaccharides-LP2 on nitric oxide production and cytokines

The occurrence of immune regulation is closely associated with the expression of cytokines such as NO, IL-6, TNF-α, and TNF-γ, etc. We detected the expression of EPS-LP2 on the levels of three cytokines in RAW264.7. The levels of NO (Figure 5B), IL-6 (Figure 5C), and TNF-α (Figure 5D) as shown in Figures 5B–D. The results demonstrated that after treatment with various concentrations of EPS-LP2. The levels of cytokines were reduced in a series of dyad-dependent ways. Still, the levels of NO and IL-6 exceeded this range. Cytokine levels were not related to EPS-LP2 concentration.

#### Impact of exopolysaccharides-LP2 on mitogen-activated protein kinase (MAPK) signals channels

**FIGURE 6:**
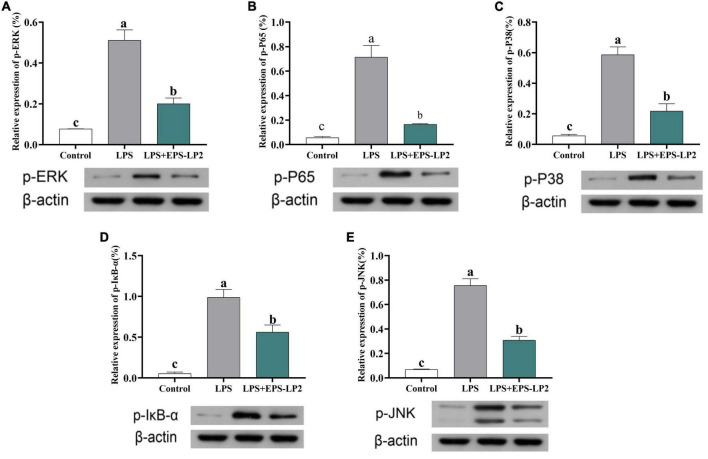
Effects of exopolysaccharides (EPS)-LP2 on the MAPK pathway **(A–C)** in RAW 264.7 cells; effects of EPS-LP2 on the NF-κB pathway **(D,E)** in RAW 264.7 cells. Different letters represent different significance from the control group (*p* < 0.05).

MAPK was considered to be one of the dominant signaling pathways in regulating pathways that regulate immune cytokines. Next, we further investigated the changes in phosphorylation levels of EPS-LP2 on three significant members of ERK, JNK, and p38 in the MAPK signaling pathway. As shown in Figures 6A–C, the level of phosphorylation of ERK, JNK, and p38 was significantly elevated in LPS-mediated cells (*p* < 0.05). LPS activated MAPK signaling pathway to phosphorylate ERK, JNK, and p38, thereby inducing immune regulation of cells. However, after treatment of cells with the same concentration of EPS-LP2, the phosphorylation levels of ERK, JNK, and p38 exceeded the blank control and negative control levels (*p* < 0.05).

#### Impact of exopolysaccharides-LP2 on nuclear factor-κB-gene binding (NF-κB) signals channels

NF-κB, as an important transcription regulator that mediates inflammation, participates in the inflammation and immune response of the body. In normal cells, the phosphorylation degree of IκB-α was significantly higher in LPS-induced cells, and the total NF-κB subunit (p65) was markedly higher. As shown in Figures 6D,E, the phosphorylation level of IκB-α was markedly reduced, and the level of phosphorylation of p65 was markedly lower in EPS-LP2-treated cells compared to LPS-induced cells.

## Discussion

**FIGURE 7:**
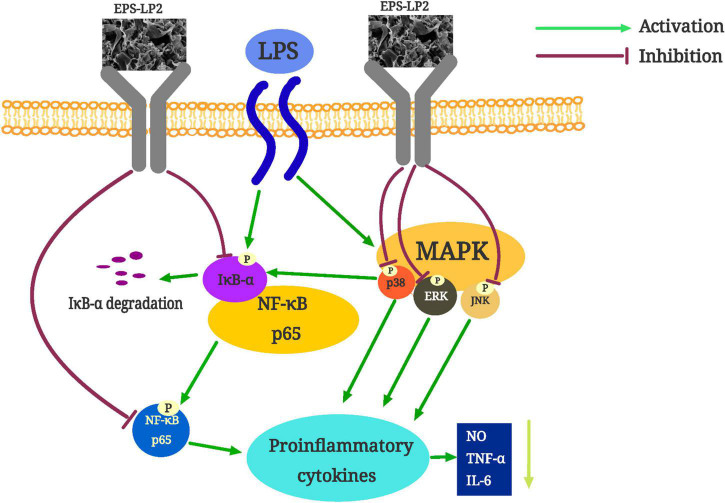
The possible mechanism of exopolysaccharides (EPS)-LP2 inhibiting inflammation in RAW 264.7 cells.

Oxidative stress is a state of unbalance between the generation of free radicals and the scavenging of antioxidant defense systems inside the body, characterized by damage to biomolecules such as proteins and nucleic acids in the cells of the body tissues (24). Induced by hydrogen peroxide, cells produce large amounts of the oxidant ROS, which converts lipid components of the cell membrane into lipid peroxides. Subsequently, lipid peroxides are degraded to MDA (25). Simultaneously, with cell membrane destruction, a large amount of lactate dehydrogenase overflows from cells. Under the condition that EPS-LP2 exists, the levels of ROS, MDA, and LDH in the body are reduced dose-dependent, as shown in Figure 7, and EPS-LP2 protects cells from oxidative stress. Enzymes ampersand non-enzymatic substances in the body assume the role of scavenging excess reactive oxygen species in the antioxidant defense system. SOD, CAT, and GSH-Px in the enzymatic antioxidant system are essential to maintaining oxidative homeostasis in the body (26). Superoxide anion radicals are turned into hydrogen peroxide by SOD during disproportionation, and then GSH-Px and CAT combine to break down the hydrogen peroxide into non-toxic substances (27). GSH is an essential Antioxidant in non-enzymatic antioxidant systems with direct biological activities and direct biological activity in scavenging free radicals (28). To penetrate and elucidate the antioxidant mechanism against the oxidation of EPS-LP2, we investigated EPS-LP2 on SOD, GSH-Px, and CAT bioactivity levels, as well as the level of GSH in cells. EPS-LP2 significantly increased the activity of SOD, GSH-Px, and CAT in cells, similar to that of Flavopiridium polysaccharide (29). Also, EPS-LP2 showed an excellent ability to increase GSH levels. Thus, EPS-LP2 improved the enzymatic antioxidant capacity of cells and reduced the damage to cells by enhancing the non-enzymatic antioxidant system.

Within the concentration range of 0–400 μg/ml, EPS-LP2 effectively inhibited NO production and inflammatory cytokines such as TNF-α, interferon γ (INF-γ), IL-1β, and IL-6 expression. This paper reported the inflammation-like immune activity of an EPS-LP2 isolated and purified from *L. plantarum*. NF-κB is a critical transcription factor of the immune signaling pathway and is associated with various human diseases, including autoimmunity, lymphoproliferative, atopic, and inflammatory diseases (30). To identify the effect of EPS-LP2 on the NF-κB signaling pathway, we determined the expression levels of p-IκB-α and p-p65. The phosphorylation levels of IκB-α and p65 were dramatically augmented in cells after LPS induction. In contrast, the phosphorylation levels of IκB-α and p65 were reduced considerably after EPS-LP2 treatment of cells. As shown in the Figure 6, it is clear that EPS-LP2 could play a role in the NF-κB signaling pathway by protecting the degradation of IκB-α and suppressing the phosphorylation of IκB-α and p65.

MAPK is an essential upstream pathway in the development of inflammation, transmitting extra-cellular signals to the cytoplasm and nucleus and regulating various biological processes. ERK, JNK, and p38 were included in the MAPK family, where ERK, JNK, and P38 were essential integrators of inflammation-inducing signals associated with human diseases such as cancer, immune disorders, inflammation, and neurodegenerative diseases (31). The phosphorylation of MAPK assumes a key role in releasing pro-inflammatory mediators and inflammatory cytokines during the inflammatory response. We continued investigating the effect of EPS-LP2 on the phosphorylation levels of ERK, JNK, and P38 belonging to MAPK in LPS-induced cells. The phosphorylation levels of ERK, JNK, and p38 were significantly reduced in LPS-induced cells after EPS-LP2 treatment (Figure 6), indicating that EPS-LP2 achieves anti-inflammatory results by repressing MAPK phosphorylation.

The results of EPS-LP2, an acidic extracellular polysaccharide with very high molecular weight and high mannose content, were thoroughly studied and analyzed. The monosaccharide composition of EPS-LP2 consisted of fucose (0.13%), arabinose (0.69%), galactose (8.32%), glucose (27.57), mannose (62.07%), fructose (0.58%) and galacturonic acid (0.46%). The monosaccharide composition and glycosidic bond types had the composition of monosaccharides and the type of glycosidic glycosyl bonds greatly impact the bioactivity of polysaccharides, such as antioxidant and immune activities (32). A mannose-rich polysaccharide has a favorable effect on preventing H_2_O_2_-induced oxidative stress in RAW264.7 cells and has some anti-inflammatory activity (33). It has now been shown that high molecular weight polysaccharides has activity (34). For example, both high (2,179,700 g/mol) and low (69,700 g/mol) molecular-weight oat β-glucans are more active, while high molecular-weight glucans are more active (35). Thus EPS-LP2, with a molecular weight of 61,260 Da, has good antioxidant and immune activity, which may be related to its essential monosaccharide component and higher molecular weight.

Generally, EPS-LP2 could enhance the activity of enzymatic oxidation and the number of antioxidant substances to protect against oxidative damage. Meanwhile, EPS-LP2 inhibits the intracellular MAPK (JNK)/NF-κB signaling pathway, reducing inflammatory factors. These abilities to cause alterations in normal cellular mechanisms may be intimately related to the specific monosaccharide composition, higher mannose content and molecular structure of EPS-LP2.

## Conclusion

An acidic extracellular polysaccharide, EPS-LP2, was extracted and purified from *L. plantarum* DMDL 9010. EPS-LP2 characteristics have been elucidated, including its molecular weight, microcosmic morphology, monosaccharides composition, major functional groups, and NMR spectrum. EPS-LP2 was composed of glycosidic bonds, including t-Man*p*, t-Glc*p*, 2-Man*p*, 6-Gal*p*, 6-Glc*p*, and 4-Glc*p*. EPS-LP2 samples were presented with irregular lamellar structures and good thermal stability. The results of H_2_O_2_-induced oxidative stress experiments were shown that the intracellular antioxidant enzyme activity EPS-LP2 could be increased and was having the effect of protecting cells from oxidative damage. At the same time, the reduction of antioxidant substances was alleviated by EPS-LP2, and the non-enzymatic antioxidant system of cells was enhanced. EPS-LP2 activated phosphorylation of ERK, JNK, and p38 in the MAPK signaling pathway. However, the phosphorylation levels of p-IκB-α and NF-κB submit (p65) in the NF-κB signaling pathway were reduced, and the stories of pro-inflammatory cytokines were reduced. In conclusion, EPS-LP2 intake was a promising prebiotic intervention strategy for the Intervention of inflammatory factors.

## Data availability statement

The original contributions presented in this study are included in the article/supplementary material, further inquiries can be directed to the corresponding author.

## Author contributions

J-MW, W-TW, J-WL, Y-TL, and Z-ZH: writing—original draft. X-ZJ: writing—review and editing; D-ML: funding acquisition. Y-YH: investigation, formal analysis, writing—review and editing, funding acquisition, and supervision. All authors contributed to the article and approved the submitted version.
